# The role of noncoding RNAs in the osteogenic differentiation of human periodontal ligament-derived cells

**DOI:** 10.1016/j.ncrna.2022.11.003

**Published:** 2022-11-14

**Authors:** Albert Sufianov, Aferin Beilerli, Sema Begliarzade, Tatiana Ilyasova, Valentin Kudriashov, Yanchao Liang, Ozal Beylerli

**Affiliations:** a Educational and Scientific Institute of Neurosurgery, Рeoples’ Friendship University of Russia (RUDN University), Moscow, Russia; b Department of Neurosurgery, Sechenov First Moscow State Medical University (Sechenov University), Moscow, Russia; c Department of Obstetrics and Gynecology, Tyumen State Medical University, 54 Odesskaya Street, 625023, Tyumen, Russia; d Republican Clinical Perinatal Center, Ufa, Republic of Bashkortostan, 450106, Russia; e Department of Internal Diseases, Bashkir State Medical University, Ufa, Republic of Bashkortostan, 450008, Russia; f Gastric Cancer Center, West China Hospital of Sichuan University, China; g Department of Neurosurgery, The First Affiliated Hospital of Harbin Medical University, Harbin, 150001, China

**Keywords:** microRNA, Long non-coding RNA, Circular RNA, Periodontal ligament cell, Periodontal ligament stem cell, Osteogenic differentiation

## Abstract

Human periodontal ligament-derived cells are important seed cells for periodontal regeneration, and their osteogenic potential closely affects alveolar bone repair and periodontal regeneration. Human periodontal ligament stem cells are pluripotent stem cells of mesenchymal origin, which can differentiate in osteoblasts and cementoblasts. However, the molecular mechanism of this differentiation activity is poorly studied. Noncoding RNAs (ncRNAs) belong to RNAs, which do not encode proteins and represent a large segment of the human transcriptome, mainly including microRNAs (miRNAs), long noncoding RNAs (lncRNAs), and circular RNAs (circRNAs). It was shown that ncRNAs is involved in the proliferation and differentiation of cells, epigenetic modifications, apoptosis, as well as in complex control and pathogenesis of various diseases. NcRNAs are actively involved in the regulation of osteogenic genes in human periodontal ligament-derived cells. This article reviews the research progress of ncRNAs in the regulatory targets, pathways and functions of ncRNAs in the osteogenic differentiation of human periodontal ligament-derived cells.

## 1 Introduction

Periodontal disease is a chronic inflammatory disease characterized by the loss of periodontal supporting tissue, and adult patients often suffer from tooth loss due to severe periodontal disease [1]. The ultimate goal of periodontal therapy is periodontal tissue regeneration, and the use of beneficial seed cells to regenerate damaged tissue is expected to be an effective way to solve this problem. Human periodontal ligament-derived cells are important seed cells for periodontal regeneration, and their osteogenic potential closely affects alveolar bone repair and periodontal regeneration. Periodontal ligament cells (PDLC) and periodontal ligament stem cells (PDLSC) in human periodontal ligament-derived cells have been reported to play an important role in periodontal regeneration as seed cells. PDLC is a heterogeneous mixed cell population derived from periodontal tissue, and its progeny can proliferate to produce fibroblasts, osteoblasts and cementoblasts, which are closely related to the reconstruction and regeneration of periodontal defects. PDLSCs are adult stem cells derived from periodontal ligament. They have excellent self-renewal and multi-directional differentiation capabilities. They can form a structure similar to natural cementum-periodontal ligament-alveolar bone complex in tissue morphology and spatial arrangement and have a huge periodontal regeneration potential [[2], [3], [4]]. Agrafioti P. et al. Indicated divergent expression profiles in macrophages driven by transcriptional regulators CIITA, RELA, RFX5, and RUNX2. Macrophages in periodontitis expressed both pro-inflammatory and anti-inflammatory markers and their polarization was not mutually exclusive. The majority of macrophages in periodontitis expressed the monocyte lineage marker CD14, indicating their bone marrow lineage. We also found high expression and activation of RELA, a subunit of the NF-κB transcription factor complex, in gingival macrophages of periodontitis patients with T2DM [5] (see Table 1).

**Table 1:** Differential miRNA expression profiles of human PDLC and affecting on pathway expression.

miRNA	expression	pathway	Reference
**MiR-146a**	down-regulated	TLR, TRAF6	25–28
		NF-κB	
		TRAF6/p38	
		MAPK	
**MiR-17∼92 family**	up-regulated	BMP	30
		Runx2	
		Smad1 and Smad5	
**MiR-21**	up-regulated	ERK-MAPK	37–40
		FGF, ACVR2B, SPRY, Runx2	
		BMP	
**MiR-214**	up-regulated	β-catenin	44
		Wnt	
**MiR-125b**	up-regulated	NKIRAS2	49
**miR-543**	up-regulated	ERBB2 2 (TOB2),	52–54
**miR-2861**	up-regulated	RUNX2	54
**miR-218**	up-regulated	RUNX2	55
**miR-1305**	up-regulated	RUNX2	56
**miR-29 family**	up-regulated	Wnt	60
**miR-132**	up-regulated	(PI3–K)/(PKB)/mTOR)	61
**miR-195-5p**	down-regulated	Wnt-3a, FGF2, BMPR1A	62

Non-coding RNAs (ncRNAs) are a class of RNAs without protein-coding functions that regulate gene expression and cell differentiation at the genomic and chromosomal levels [[6], [7], [8], [9]]. According to its length, it is mainly divided into two categories, short-chain ncRNA (such as microRNA (microRNA, miRNA), etc.) and long-chain ncRNA (long non-coding RNA, lncRNA). Circular RNAs (circRNAs) are a special class of ncRNAs that are different from linear RNAs [10]. Recent studies have found that there is a close relationship between periodontal disease and ncRNA, and ncRNA is actively involved in the regulation of osteogenic genes in human periodontal ligament-derived cells. Scholars screened out 159 miRNAs and 8925 lncRNAs with differential expression between periodontitis gingival tissue and healthy gingival tissue, indicating that ncRNAs may be potential targets for periodontitis treatment, and may be a potential target for human periodontal disease [11,12]. Membrane-derived cell-mediated periodontal regeneration provides guidance. Alveolar bone regeneration is a key and important link in periodontal regeneration. Elucidating the role of ncRNA network in the osteogenic differentiation of PDLC/PDLSC can help to better regulate the expression of osteogenic genes and promote the development of periodontal regeneration treatment strategies.

## 2 Potential relationship between lncrnas and osteogenic differentiation of human periodontal ligament-derived cells

The results of human whole-genome sequencing showed that ncRNAs without protein-coding functions accounted for more than 98% of the entire transcripts, indicating that ncRNAs may play an important role in human biology to regulate gene expression and determine the fate of cell differentiation. Studies found that ncRNAs regulate the differentiation of a variety of human mesenchymal stem cells, thereby participating in the osteogenesis process [10,13].

In the field of periodontal regeneration, the potential relationship between ncRNAs and the osteogenic differentiation of human periodontal ligament-derived cells has received increasing attention. With the rapid development of high-throughput detection technology and bioinformatics analysis methods, a variety of ncRNAs that may play a role in the osteogenic differentiation of human periodontal ligament-derived cells have been identified.

Zhang et al. reported 149 upregulated and 169 downregulated lncRNAs in differentiated pPDLSCs compared with their expression states in healthy PDLSCs [14]. These lncRNAs are implicated in the oxidative phosphorylation which play significant roles in both osteogenic differentiation and inflammation, as well as the inflammatory processes such as IL-2 signaling and IL-8 signaling. Additionally, MEG3, pPDLSC osteogenesis impairment-related lncRNA (POIR), TWIST1, and hypoxia-inducible factor-1α antisense RNA 2 (HIF1A-AS2) also participate in the osteogenic differentiation of pPDLSCs [[15], [16], [17]]. MEG3 is an imprinted gene involved in mesenchymal stem cell differentiation [18]. In periodontitis, downregulation of MEG3 suppressed the osteogenic differentiation of pPDLSCs through the miR-27a3p/IGF1 axis and the PI3K/Akt signaling pathway [18]. Similarly, POIR was significantly downregulated in pPDLSCs compared to hPDLSCs, and knockdown of it negatively regulated osteogenic differentiation of pPDLSCs by sponging miR-182 and depressing the expression of FoxO1 [5]. Because FoxO1 could increase bone formation in pPDLSCs by competing with T-cell factor 4 for β-catenin and inhibiting the canonical Wnt pathway. Besides, lncRNA-TWIST1 was also markedly downregulated in pPDLSCs. Further research suggested that lncRNA-TWIST1 overexpression could promote the osteogenic differentiation of pPDLSCs by inhibiting TWIST1 mRNA expression and activating the Wnt/β-catenin signaling pathway [18].

## 3 The role of mirnas in the osteogenic differentiation of human periodontal ligament-derived cells

**Fig. 1:**
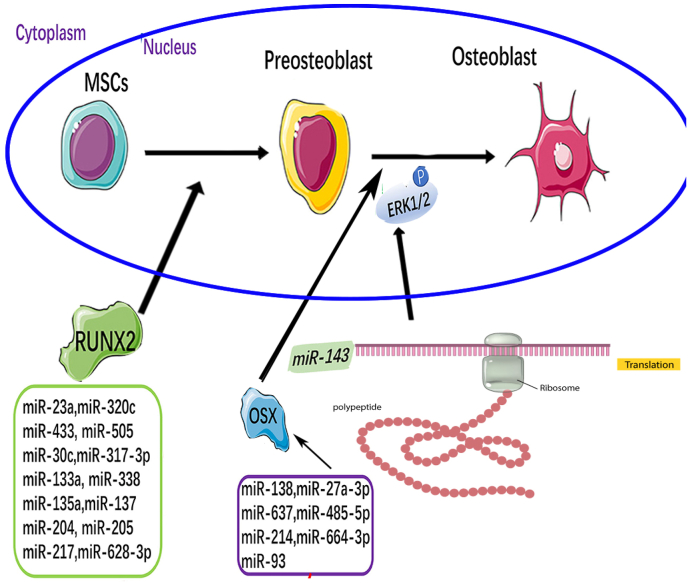
The role of various microRNAs in osteogenic differentiation of human PDLSCs.

MiRNAs is a class of single-stranded small molecule non-coding transcripts of about 20 nucleotides in length. Mature miRNAs induce mRNA degradation or inhibition by specifically pairing with the 3′-untranslated region (UTR) of target gene mRNAs. Its translation initiation, negatively regulates gene expression, and affects cell differentiation, proliferation and apoptosis at the post-transcriptional level [10]. During the differentiation of human periodontal ligament-derived cells exposed to different osteogenic conditions, miRNAs play an indispensable regulatory role by targeting osteogenic markers or osteogenesis-related pathways (Fig. 1).

Chang et al. have identified 32 miRNAs and 818 mRNAs differentially expressed under tensile force-induced osteogenic differentiation of PDLC [19]. In the study of PDLSC, Hao et al. found that 116 miRNAs were differentially changed after the induction of human PDLSC mineralization, and 31 miRNAs were predicted to have osteogenesis-related target genes [20]. Qu et al. found that 2171 lncRNAs and 3557 messenger RNAs (mRNAs) were differentially expressed after mineralization induction in human PDLSCs, of which 393 lncRNAs were closely related to osteogenesis-related mRNAs, which were predicted to be involved in mitogen activation Protein kinase (mitogen-activated protein kinase, MAPK), vascular endothelial growth factor (vascular endothelial growth factor, VEGF) and transforming growth factor (transforming growth factor, TGF)-β and other signaling pathways [21]. Huang et al. stimulated PDLSCs with 2 g*cm-2 compressive stress for 12 h, 90 lncRNAs were differentially expressed, 72 were up-regulated and 18 were down-regulated [22]. Zheng et al. revealed that the osteogenic differentiation of human PDLSCs is different at the same time, 8 circRNAs were down-regulated, 44 circRNAs were up-regulated, 4 miRNAs were up-regulated, and 15 miRNAs were down-regulated, thus constructing a regulatory network including 50 circRNAs, 55 miRNAs, and 613 mRNAs [23]. Wang et al. successfully constructed a circRNA-miRNA network differentially expressed in the osteogenic differentiation of human PDLSCs loaded with tensile force [24]. The above studies show that many ncRNAs can undergo significant changes in response to mechanical stress and mineralization induction, and are highly correlated with changes in osteogenesis-related gene mRNAs.

### 3.1 MiR-146a

MiR-146a is one of the early identified miRNAs involved in the regulation of inflammation, which can negatively regulate the Toll-like receptor (TLR) signaling pathway related to the pathogenesis of inflammation, tumor necrosis factor receptor-related factor 6 (tumor necrosis factor 6) -receptor association factor 6, TRAF6) is a key adaptor molecule for this pathway and has been shown to be a target of miR-146a. Inflammatory stimulators such as lipopolysaccharide (LPS), tumor necrosis factor (TNF)-α, etc. activate TRAF6, which in turn activate downstream nuclear factor (NF)-κB, p38 MAPK and extracellular regulation Protein kinases (ERK). In addition, the regulation of bone differentiation by NF-κB is also quite important [25].

In human PDLC, miR-146a downregulates the NF-κB pathway and the TRAF6/p38 MAPK signaling pathway, and negatively regulates the secretion of pro-inflammatory factors [26,27]. After exposure to LPS-stimulated human PDLCs treated with miR-146a mimics, the expression of osteogenic markers was up-regulated and the inflammatory NF-κB p65 activity was inhibited, thereby reversing the inhibition of LPS-induced osteogenic differentiation of PDLCs [28].

### 3.2 MiR-17–92 family

MiR-17 is a core member of the miR-17–92 family, which plays a key role in regulating cell proliferation and differentiation and controlling cell cycle progression [29]. In an inflammatory microenvironment with elevated TNF-α levels, the expression of Smad ubiquitin regulatory factor 1 (Smurf1), an important negative regulator of osteogenic differentiation, is upregulated, and its upregulation will degrade bone morphogenesis protein (BMP), Runt-related transcription factor 2 (Runx2), Smad1 and Smad5 [30].

A study found that TNF-α, miR-17 and Smurf jointly regulated the osteogenic differentiation of PDLSCs [29]. In healthy tissue-derived PDLSCs, down-regulated miR-17 promoted osteogenesis, while in an inflammatory microenvironment, down-regulated miR-17 promoted osteogenesis. MiR-17 increased the expression of Smurf and inhibited osteogenesis, indicating that miR-17 played a biphasic role in the osteogenic differentiation of PDLSC in different microenvironments.

The relationship between the Wnt canonical pathway and different cultured microenvironments resembles a biphasic network. Human Wnt-3a protein down-regulates miR-17 in complete medium and up-regulates miR-17 in osteogenic differentiation medium. Different expression levels of miR-17 target the key transcriptional factor 3 (TCF3) of the Wnt pathway, reversing the positive or negative regulation of PDLSC osteogenesis by the Wnt pathway. That is, miR-17 can act as a sensitive "toggle switch" that rapidly responds to Wnt signals in different microenvironments [31].

As a member of the miR-17–92 family, miR-20a can promote osteogenesis of human bone marrow mesenchymal stem cells through the BMP signaling pathway [32]. Scholars found through transient transfection that miR-20a also promoted the osteogenic differentiation of human inflammatory PDLSCs and confirmed that its upstream histone deacetylase (histone deacetylase, HDAC) 9 inhibited the expression of miR-17–92 family at the transcriptional level [33,34]. Another study used advanced glycation end products (AGEs) as stimuli, and the results showed that the osteogenesis of human PDLSC was inhibited, miR-17 was down-regulated, and the Wnt canonical pathway was activated, suggesting that miR-17 is a potential target for the treatment of diabetic periodontitis [35,36].

### 3.3 MiR-21

MiR-21 is an embryonic stem cell differentiation-related miRNA that functions through self-maintenance under different physiological and pathological conditions [37].

Sprouty family members are a class of proteins necessary for stem cell differentiation. Sprouty1 (Spry1) is a target gene of miR-21 and participates in and negatively regulates ERK-MAPK and fibroblast growth factor (FGF) signaling pathways. It has been shown to promote osteogenesis of mesenchymal stem cells [38,39]. Research showed that miR-21 expression was up-regulated during normal PDLSC osteogenic differentiation, while miR-21 expression was down-regulated in an inflammatory microenvironment, and Spry1 expression was inhibited after treatment with the inflammatory factor TNF-α, indicating that TNF-α targets osteogenic differentiation of PDLSCs and inhibit towards the miR-21/Spry axis [37].

As an upstream regulator, Smad5 promotes the expression of osteogenic markers such as Runx2 and mediates the BMP signaling pathway [30]. A study showed that miR-21 overexpression in osteogenic-induced PDLSC mediates osteogenic differentiation by directly targeting Smad5 [40].

Periodontal ligament associated protein (PLAP)-1 is a negative regulator that affects periodontal osteogenesis homeostasis and is specifically expressed during PDLC differentiation. Li et al. found that the expression level of PLAP-1 was negatively correlated with miR-21 and miR-101 during PDLC osteogenic differentiation, confirming that two miRNAs target PLAP-1 to regulate PDLC osteogenesis [41]. In addition, miR-21 can act as a key miRNA in response to stretching force and increase its expression, targeting activin receptor type IIB (ACVR2B), which is crucial in the activation of activin and is an important regulator of the TGF-β pathway. Its downregulation is beneficial to mediate the osteogenic differentiation effect of PDLSC [42].

### 3.4 MiR-214

MiR-214 directly targets activating transcription factor 4 (ATF4) to inhibit osteoblast activity [43]. ATF4 is a key regulator of osteogenic differentiation and can induce osteoblast differentiation by upregulating the level of β-catenin (β-catenin) in the Wnt canonical signaling pathway [44].

MiR-214 also showed similar osteogenic inhibitory effect in human PDLSC, significantly down-regulated miR-214 inhibited ATF4 by binding, or targeting the 3′-UTR of β-catenin gene CTNNB1 to inhibit Wnt/β-catenin signaling pathway, which in turn negatively regulates the osteogenic differentiation of PDLSCs [45,46].

### 3.5 MiR-125b

MiR-125b, an early-discovered miRNA involved in bone development and remodeling, plays a key role in inhibiting osteogenic differentiation of mesenchymal stem cells [47].

Inhibition of NF-κB signaling can effectively enhance osteogenic differentiation, and NF-κB inhibitor interacting RAS-like 2 (NKIRAS2) is a target gene of miR-125b [48]. miR-125b negatively regulates NKIRAS2 expression, activates NF-κB signaling, and inhibits PDLC osteogenic differentiation, suggesting that therapeutic inhibition of miR-125b can promote osteogenesis and may even partially reverse the progression of periodontitis [49].

The regulation of connexin 43 (Cx43) can regulate intercellular communication, thereby regulating cell proliferation and differentiation. The expression of Cx43 is increased during tooth formation in mice and is involved in the regulation of tooth mineralization [50]. Recently was found, that transfection of miR-125b in human PDLSCs can inhibit PDLSC osteogenesis by targeting Cx43 [51].

### 3.6 Other miRNAs that respond to mineralization induction

The expression of miR-543 and miR-22 was significantly increased after osteogenic induction of human PDLSC, and the up-regulated miR-543 negatively targeted the transducer of ERBB2 2 (TOB2), and miR-22 targeted histones deacetylase (HDAC)6, promotes the osteogenic differentiation of PDLSC [[52], [53], [54]]. After inoculation of human PDLSCs on three-dimensional scaffolds for induction of mineralization, miR-2861 up-regulated the expression of Runx2 protein, showing superior osteogenicity compared to the scaffold-free group [54]. miR-218 expression was significantly down-regulated during osteogenic induction of human PDLSCs, dental pulp stem cells, and gingival stem cells; miR-1305 was up-regulated after osteogenic induction of human PDLSCs exposed to nicotine; Runx2 was patway for miR-218/miR-1305. The high expression of miR-218/miR-1305 negatively regulates the expression of Runx2 and inhibits the osteogenic differentiation potential of human PDLSC. miR-1305 may provide new insights into the potential diagnosis and treatment targets of periodontal disease in smoking population [55,56].

In addition to mineralization induction, exogenous mechanical stress stimulation can also affect the osteogenic differentiation of human periodontal ligament-derived cells and play an important role in the dynamic balance of alveolar bone remodeling. Stresses include fluid shear, tensile and compressive stresses.

### 3.7 The miR-29 family and other miRNAs that respond to mechanical forces

The miR-29 family members include miR-29a, miR-29b and miR-29c, which can not only regulate the Wnt pathway to promote osteogenesis, but also inhibit the synthesis of osteonectin and the Notch pathway to inhibit osteogenesis [57].

MiRNAs play important roles in tissue development and remodeling by regulating extracellular matrix (ECM) gene clusters. During orthodontic loading, the expression of major periodontal ECM genes (such as Col1A1, Col3A1 and Col5A1 encoding the α1 chain of collagen types I, III, and V) is increased on the tension side and decreased on the pressure side, which is essential for tooth movement due to bone remodeling [58,59]. MiR-29 family members are up- and down-regulated under cyclic tensile and compressive forces, acting as regulators of ECM homeostasis during chewing or orthodontic tooth movement by directly targeting the ECM genes Col1A1, Col3A1, and Col5A1, affecting PDLC formation. bone [60].

Studies have confirmed the central role of miR-132 in cell differentiation. Qi et al. used fluid shear force to induce osteogenic differentiation of PDLC [61]. Compared with the untreated group, miR-132 was significantly up-regulated, indicating its specific role in osteogenic differentiation. In addition, miR-132 can activate the canonical phosphoinositide 3-kinase (PI3–K)/protein kinase B (PKB)/mammalian target of rapamycin rapamycin, mTOR) signaling axis, regulates the osteogenic differentiation of human PDLC induced by fluid shear stress. Controlling the balance between miR-132 and mTOR signaling pathways in PDLC may potentially affect periodontal regeneration.

Chang et al. analyzed the differential miRNA-mRNA expression profiles of human PDLC osteogenic differentiation induced by tensile force, and speculated that miR-195-5p, miR-424-5p, miR-1297, miR-3607-5p, miR-145 -5p, miR-4328 and miR-224-5p may act as core miRNAs and play key regulatory roles in the osteogenic differentiation of PDLC [19]. Among them, miR-195-5p was confirmed to be significantly downregulated in response to tensile stress and negatively correlated with osteogenic differentiation, and mechanosensitive miR-195-5p directly targeted Wnt-3a, FGF2 and BMP receptor-1A (bone morphogenetic protein receptor-1A, BMPR1A), inhibits the osteogenic differentiation of PDLC [62].

During the osteogenic differentiation of periodontal ligament-derived cells, many miRNAs can be significantly changed in response to the induction of mineralization or mechanical force, target related osteogenic genes or related pathways, and play an important regulatory role that cannot be ignored.

## 4 The role of lncrnas in the osteogenic differentiation of human periodontal ligament-derived cells

### 4.1 Post-transcriptional regulation

In recent years, competing endogenous RNAs (ceRNAs) have gradually emerged as new players in the miRNA-target gene loop. CeRNAs competitively bind miRNAs through miRNA response elements, affecting gene silencing caused by miRNAs, and many lncRNAs have been shown to regulate osteogenesis at the post-transcriptional level as ceRNAs [13].

Anti-differentiation noncoding RNA (ANCR), one of the newly discovered lncRNAs, is downregulated during stem cell differentiation [63]. Down-regulated ANCR can activate the Wnt pathway and promote the osteogenic differentiation of PDLSCs [64]. ANCR has been shown to function as a miR-758 sponge, miR-758 targets Notch2 to inhibit the Notch2-Wnt/β-catenin pathway, and overexpressed ANCR inhibits PDLSC osteogenesis through the lncRNA ANCR/miR-758/Notch2 axis [65].

PCAT1 is a lncRNA associated with osteosarcoma proliferation, invasion and metastasis [66]. lncRNA-PCAT1 can act as a ceRNA to bind miR-106-5p, reverse its inhibitory effect of targeting BMP-2 and Smad4, and promote PDLSC osteogenic differentiation [67].

A study reported that the newly discovered lncRNA ENST00000446358 (lncRNA-POIR) in PDLSC of patients with periodontitis mutually inhibited miR-182 and regulated the target gene of miR-182, forkhead box protein O1 (FoxO1), Inhibit the Wnt pathway, thereby enhancing the osteogenic effect [68]. Furthermore, aberrant activation of the NF-κB pathway during inflammation upregulated miR-182 expression, leading to dysregulation of the lncRNAPOIR-miR-182 network, affecting the osteogenic differentiation of human PDLSCs.

Zou Wanhua et al. up-regulated and down-regulated the expression of lncRNA linc-01135 in inflammatory PDLSCs by lentivirus, and linc-01135 could be used as the ceRNA of miR-17-5p and miR-106b-5p to promote the growth of miR-17-5p under 12% tensile bone force [69].

Zhang et al. integrated the lncRNA expression profile of PDLSC osteogenic differentiation, and the cell subset was composed of undifferentiated PDLSC (uPDLSC), differentiated PDLSC without TNF-α stimulation (dPDLSC)) and differentiated PDLSCs under TNF-α stimulation (TNF-α-dPDLSCs), 63 lncRNAs were identified to be highly expressed in the PDLSC population; 407 lncRNAs were found to be differentially expressed between uPDLSCs and dPDLSCs, uPDLSCs; 318 lncRNAs were found to be differentially expressed between TNF-α-PDLSCs and TNF-α-PDLSCs [70]. Also they revealed the complex mechanism of lncRNAs acting as ceRNA-binding miRNAs to indirectly regulate mRNAs, and discovered 464 competing lncRNA-mRNAs, which show a many-to-many relationship and tend to be remote-regulated.

### 4.2 Transcriptional level regulation

LncRNAs can directly affect the transcription of target genes, or regulate gene transcription by regulating the activity of corresponding proteins [13].

Research found that in the time course of human PDLSC osteogenic differentiation, the up-regulated lncRNAs MEG8 and MIR22HG were highly consistent with the temporal expression patterns of four osteogenic mRNAs, namely COL6A1, which encodes the α1 chain of collagen VI, VCAN, ribosome-binding protein 1 (RRBP1) and cAMP responsive element binding protein 3 like 1 (CREB3L1), high expression of MEG8 and MIR22HG were significantly up-regulated [71]. The expression of osteogenic markers indicates that it has the potential to promote osteogenic differentiation.

Maternally expressed gene 3 (MEG3), a tumor suppressor gene that promotes osteogenic differentiation of mesenchymal stem cells, is down-regulated after human PDLC osteogenic differentiation, and overexpressed lncRNA MEG3 competes with BMP2 mRNA Heterogeneous nuclear ribonucleoprotein I (hnRNP I) inhibits the osteogenic differentiation of PDLC [72,73].

He et al. found that the lncRNA taurine upregulated gene 1 (TUG1), which was significantly up-regulated in osteogenic induction, targeted the lin-28 homolog A Lin28A, and the two positively regulated each other to promote PDLSC osteogenesis [74].

Xu Yuerong et al. used lentivirus to up-regulate and down-regulate lncRNA-7460 in inflammatory PDLSCs and found that lncRNA-7460 promotes osteogenesis in vitro, but the mechanism of action has not been fully elucidated [75].

## 5 The role of circrna in the osteogenic differentiation of human periodontal ligament-derived cells

Gu et al. identified 147 lncRNAs, 1382 circRNAs combined with 148 miRNAs in PDLSC osteogenic differentiation, and competed with 744 mRNAs for miRNA binding sites [76]. Among them, lncRNAs TCONS_00212979, TCONS_00212984, circRNA BANP, and circRNA ITCH interact with miR-34a and miR-146a, respectively, and it is predicted that miR-34a targets dual specificity protein phosphatase 1 (DUSP1) and is related to apoptosis. Factor associated suicide (FAS) and Ras-related C3 botulinum toxin substrate 1 (RAC1), miR-146a targets platelet-derived growth factor receptor alpha polypeptide (platelet-derived growth factor receptor alpha polypeptide) growth factor receptor alpha, PDGFRA), TGF-β receptor 2 (transforming growth factor beta receptor II, TGFBR2) and Myc, regulate PDLSC osteogenic differentiation through the MAPK pathway.

Antisense cerebellar degeneration-related protein 1 transcript (CDR1as) has about 70 conserved miR-7 binding sites and performs biological functions as a miR-7 sponge [77]. Recently, CDR1as has been shown to act upstream of miR-7, inhibiting the effect of miR-7 on growth differentiation factor (GDF) 5, and up-regulated GDF5 enhances the phosphorylation of Smad1/5/8 and p38 MAPK, this in turn promotes the osteogenic differentiation of PDLSCs [78].

Under the osteogenic induction conditions of mineralization or application of mechanical force, lncRNA/circRNA-miRNA constitutes a complex but traceable important regulatory network in the osteogenic differentiation of periodontal-derived cells, providing a new strategy for periodontal regeneration.

## 6 Conclusions

MiRNAs, lncRNAs and circRNAs can all function as important regulators in the osteogenic differentiation of periodontal ligament-derived cells under mineralization and mechanical induction. miRNAs often act directly on mRNAs of osteogenic target genes; lncRNAs can either bind to mRNAs of osteogenic target genes, or serve as ceRNAs to competitively bind miRNAs to achieve transcriptional or post-transcriptional regulation; circRNAs mainly act as ceRNAs to competitively inhibit miRNAs. The three are cross-linked with each other, forming a huge and complex lncRNA/circRNA-miRNA regulatory network, which has both promoting and inhibiting effects on the osteogenic differentiation of periodontal ligament-derived cells.

Identifying the differentially expressed ncRNAs of periodontal ligament-derived cells during osteogenic differentiation and revealing their regulatory mechanisms is one of the key scientific issues in the field of periodontal regeneration molecular medicine [[79], [80], [81], [82]]. However, at present, the research on related ncRNAs, especially circRNAs, is still in its infancy, and further exploration is urgently needed to improve the periodontal regeneration RNA information database. PDLC/PDLSC osteogenesis-related ncRNAs can be used as biomarkers for the diagnosis and prognosis assessment of periodontal disease, and regulating their expression levels is expected to prospectively realize the directional osteogenic differentiation of human periodontal ligament-derived cells, which may provide a basis for the development of treatment for periodontal disease and osteogenesis. Drugs for related diseases provide candidate targets and have broad application prospects. However, the clinical translation of ncRNA-targeted drugs faces several major problems, including the effective carrier of ncRNA, chemical modification, route of administration, drug targeting, drug toxicity, etc., which still require the unremitting efforts of researchers and practical pursuit of truth. It is believed that in the near future, with the maturity of anti-inflammatory bone destruction drug development from preclinical to clinical translation, based on the ncRNA related to osteogenic differentiation of periodontal ligament-derived cells, that is, a certain or Drugs targeting multiple effector molecules will surely bring breakthrough progress in human periodontal regeneration and bone health.

## Funding

This work was supported by the Bashkir State Medical University Strategic Academic Leadership Program (PRIORITY-2030).

## Author contributions

Albert Sufianov and Sema Begliarzade conceptualized and designed the study. All authors participated in the acquisition, analysis and interpretation of the data. Valentin Kudriashov drafted the manuscript. Tatiana Ilyasova, Yanchao Liang contributed to critical revisions of the manuscript. All authors agreed on the journal to which the article would be submitted, gave final approval for the version to be published, and agreed to be accountable for all aspects of the work.

## Declaration of competing interest

The authors declare they have no conflict of interest.
